# Pregnancy-Induced Hypertension “Probably Linked” to PFOA Contamination

**DOI:** 10.1289/ehp.120-a59

**Published:** 2012-02-01

**Authors:** Wendee Holtcamp

**Affiliations:** Houston-based freelancer Wendee Holtcamp has written for *Nature*, *Scientific American*, *National Wildlife*, and other magazines.

Working as part of a class-action legal settlement with DuPont, a science panel appointed by the Wood County Circuit Court recently announced the first “probable link” between perfluorooctanoic acid (PFOA) released into the environment from a DuPont manufacturing plant and disease in neighboring communities—specifically, pregnancy-induced hypertension (PIH).^1^ The panel was ordered to return a “probable link” finding if occurrence of a disease in members of the settlement class was judged “more likely than not” to be increased by the members’ PFOA exposure. With this first “probable link” designation, the next stage of the settlement kicks in, whereby DuPont must pay up to $235 million to monitor class members for any diseases for which a probable link is determined.^2^

PFOA can be released into the environment during the manufacture of products such as Teflon™ nonstick coating. The chemical is present in an estimated 98% of Americans, with average blood serum levels of 5.2 ng/mL reported in the 1999−2000 National Health and Nutrition Examination Survey.^3^ For people living in the 6 affected Ohio and West Virginia public water districts surrounding DuPont’s Washington Works plant, serum PFOA levels averaged 32.91 ng/mL.^4^ PFOA had leached into groundwater and contaminated the Ohio River, tainting drinking water supplies.^5^

In 2004 a settlement reached between DuPont and the plaintiffs established the C8 Health Project^6^ to study health effects of PFOA exposures in communities surrounding the plant. More than 69,000 affected members of the class submitted blood samples and completed a health questionnaire during 2005 and 2006. Under the terms of the settlement, a C8 Science Panel was established to use all scientifically relevant data to determine whether PFOA is linked to human diseases. Panelists included epidemiologists Tony Fletcher, David Savitz, and Kyle Steenland.

Savitz, a professor at Brown University, explains that the panel originally envisioned using the C8 Health Project data and a literature review to report on potential health effects but soon realized that although animal studies have linked PFOA to tumor formation, neonatal death, impaired thyroid, and lipid abnormalities,^7^^,^^8^ few studies have evaluated human health effects. So the panelists generated their own research from the exposed cohort.

In December 2011 the panel submitted to the court its first probable link reports on reproductive outcomes (PIH, birth defects, miscarriage/still births, and preterm birth/low birth weight). For the PIH report, the panel relied on 6 studies examining either PIH or preeclampsia, a life-threatening condition for mother and fetus characterized by protein in the urine (proteinuria) combined with PIH. The panelists conducted 5 of the studies themselves, with collaborators.

The studies were unable to distinguish between PIH with or without proteinuria because of the data available. Because women with preeclampsia by definition have hypertension, the panelists assessed only a link between PIH and PFOA. “There were a series of studies that pointed variably and modestly towards there being an association,” Savitz says. “There were different methodological limitations in each of them, but in looking at the aggregation of them, there really is a signal there.”

Although it’s well established that PIH and preeclampsia can cause preterm birth and stillbirth, the panel concluded that the available data were insufficient to support a “probable link” between PFOA exposure and preterm birth or stillbirths/miscarriages, or birth defects, either. “We were careful not to claim that there is no impact [on these other PIH-related end points]—rather, that there is insufficient evidence to support a probable link,” says Fletcher, a professor at the London School of Hygiene & Tropical Medicine. “This may be due to clear evidence of no effect or insufficient power, and obviously the rarer the outcome, the more that low power will result in insufficient evidence to declare a link.”

The C8 Health Project is one of the largest and most comprehensive studies of its kind to result from a legal settlement. “The agreement . . . resulted in, first, a very large and rapidly collected data set by a court-appointed survey group, and then significant additions to understanding the population science related to perfluorocarbon exposure by independent researchers,” says Alan Ducatman, chairman of community medicine at West Virginia University. “These socially reasonable outcomes help . . . everyone by adding to the science and being accountable to population needs. That is a credit to the settling parties.”

The panel released 3 status reports in December, one with additional details on the reproductive outcomes,^9^ one on thyroid function,^10^ and one on serum cholesterol levels.^11^ They found a statistically significant drop in LDL (“bad”) cholesterol associated with PFOA declines in individuals following water treatment, as well as a significant increase in thyroid disease in women with higher PFOA levels. Although status reports are used in the panel’s final assessments, Fletcher stresses that “one cannot impute the upcoming probable link decision from one status report alone.” The panel expects to release its remaining “probable link” assessments by July 2012.

**Figure:**
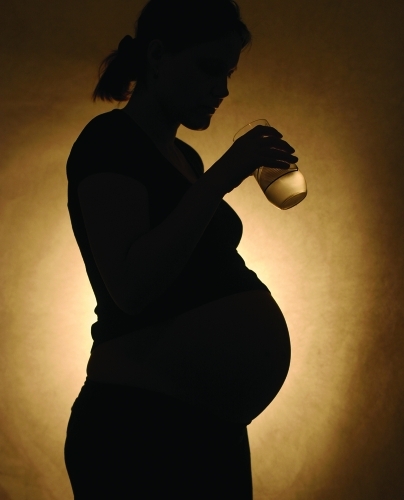
Drinking water contaminated with PFOA probably contributed to pregnancy-induced hyper­tension in women living near a DuPont plant near Parkersburg, WV.
